# Maternal nutrient restriction and the fetal left ventricle: Decreased angiotensin receptor expression

**DOI:** 10.1186/1477-7827-3-27

**Published:** 2005-07-14

**Authors:** Jeffrey S Gilbert, Alvin L Lang, Mark J Nijland

**Affiliations:** 1 Center for Pregnancy and Newborn Research, Department of Obstetrics and Gynecology, University of Texas Health Science Center at San Antonio, San Antonio, TX, USA; 2 Department of Zoology and Physiology, University of Wyoming, Laramie, WY, USA

## Abstract

**Background:**

Adequate maternal nutrition during gestation is requisite for fetal nutrition and development. While a large group of epidemiological studies indicate poor fetal nutrition increases heart disease risk and mortality in later life, little work has focused on the effects of impaired maternal nutrition on fetal heart development. We have previously shown that 50% global nutrient restriction from 28–78 days of gestation (early to mid-pregnancy; term = 147 days) in sheep at mid-gestation retards fetal growth while protecting growth of heart and results in hypertensive male offspring at nine months of age. In the present study, we evaluate LV gene transcription using RNA protection assay and real-time reverse transcriptase polymerase chain reaction, and protein expression using western blot, of VEGF and AT1 and AT2 receptors for AngII at mid-gestation in fetuses from pregnant ewes fed either 100% (C) or 50% (NR) diet during early to mid-gestation.

**Results:**

No difference between the NR (n = 6) and C (n = 6) groups was found in gene transcription of the AngII receptors. Immunoreactive AT1 (1918.4 +/- 154.2 vs. 3881.2 +/- 494.9; P < 0.01) and AT2 (1729.9 +/- 293.6 vs. 3043.3 +/- 373.2; P < 0.02) was decreased in the LV of NR fetuses compared to C fetuses. The LV of fetuses exposed to NR had greater transcription of mRNA for VEGF (5.42 ± 0.85 vs. 3.05 ± 0.19; P < 0.03) than respective C LV, while no change was observed in immunoreactive VEGF.

**Conclusion:**

The present study demonstrates that VEGF, AT1 and AT2 message and protein are not tightly coupled, pointing to post-transcriptional control points in the mid gestation NR fetus. The present data also suggest that the role of VEGF and the renin-angiotensin system receptors during conditions inducing protected cardiac growth is distinct from the role these proteins may play in normal fetal cardiac growth. The present findings may help explain epidemiological studies that indicate fetuses with low birth weight carry an increased risk of mortality from coronary and cardiovascular disease, particularly if these individuals have reduced cardiovascular reserve due to an epigenetic decrease in vascularization.

## Background

Adequate maternal nutrition is of the utmost importance for proper fetal growth and development [1,2]. Epidemiological evidence links impaired maternal nutrition and compromised fetal growth to increased incidence of cardiovascular disease in adulthood [3,4]. LV hypertrophy and increased ventricular wall thickness are generally agreed to be powerful and independent risk factors for coronary heart disease, stroke and sudden death. Despite the observations linking maternal NR and increased mortality from heart disease in adults, few studies have been published investigating the effects of maternal NR on the development of the fetal myocardium. This rings especially true in the context of the developmental origins hypothesis which describes a causal link between poor materno-fetal nutrition and cardiovascular disease in later life [5], and in which there is a marked lack of published reports examining NR-induced changes at the molecular level in the mid-gestation fetal heart.

Work from our group has reported the development of a model of global NR encompassing the first half of gestation in sheep [6]. Several aspects of this model have already been detailed at 78 dGA, including IUGR with protected heart growth (as indicated by increased LV to fetal weight ratio) [6]; altered transcription of numerous hypertrophy related genes in the fetal cardiac LV [7]; increased incidence of oxidative base lesions in fetal oogonia [8]; and altered concentrations of amino acids in fetal fluids [9]. Moreover, we have recently shown the same paradigm of global maternal NR during early to mid-gestation results in altered expression of components of the renal renin-angiotensin system and hypertension in offspring at nine months of age [10].

Studies in the rat show that IUGR induced by sodium restriction during gestation results in several alterations in gene transcripts and protein expression in hypertensive adult females [11], but there are no reports to our knowledge detailing possible contributions of alterations in fetal gene expression to the observed adult phenotype from this model. Maternal NR during late gestation in sheep has been show to affect fetal cardiovascular function by decreasing basal heart rate [12] and increasing fetal blood pressure [13], however these studies have not reported changes in gene transcription or protein expression of any proteins involved in cardiac development. Distinct patterns of mRNA and protein expression for the AngII receptors AT1 and AT2 have been noted during heart development in the sheep [14,15]. Sundgren and coworkers have demonstrated that angiotensin II (AngII) mediates hyperplastic but not hypertrophic activity in the ovine fetal heart [16]. Others have implicated the renin-angiotensin system in the processes of normal and pathological cardiac growth in both the fetus and adult [17-19]. Likewise, vascular endothelial growth factor (VEGF) is recognized as an important contributor to normal fetal growth and development [20]. Moreover, it has recently been show that VEGF and the renin-angiotensin system posses an intimate relationship regarding the promotion of angiogenesis and inflammation [21,22] and that AT1 inhibition impairs VEGF mediated coronary angiogenesis [23].

Given the association between VEGF, the renin-angiotensin system via the AT1 receptor, and protected cardiac ventricular growth introduced above, and given that we have suggested that the regulation of fetal LV growth and development following NR likely involves the two entry points – VEGF and the AT1 receptor [7], we report here the results on an investigation of the hypothesis that both VEGF and the AT1 receptor exhibit increased gene and protein expression at mid gestation in LV tissue from NR compared to control fed fetuses.

## Methods

### Animals and Experimental Groups

Animal protocols were approved by the University of Wyoming Institutional Animal Care and Use Committee. The tissues utilized in this study derive from the same cohort of animals previously published [6-9] whereby pregnant ewes were fed either a control (C: 100% of National Research Council suggested rations) or NR (50% NRC rations) from 28–78 dGA (term = 147 days). Ewes and fetuses were euthanized at 78 dGA in the manner described previously [6]. The cohort consisted of twelve fetuses from eleven pregnancies. Of the eleven pregnancies, five were NR (3 twins, 3 singletons; 4 female, 2 male) and six were C (3 twins, 3 singletons; 3 female, 3 male). When possible one fetus was chosen at random from a twin pregnancy; however in one case two males from a twin pregnancy were utilized in an attempt to maximize the number of males in the NR group.

### Tissue Collection

Fetal hearts were excised, cleansed of connective tissues, blotted dry and weights were recorded. Fetal left ventricles were dissected, quickly weighed, snap frozen and stored at -80°C for later analysis.

### Protein and RNA Extraction

Total soluble protein and RNA were extracted from the left ventricles as detailed previously [10,24].

### Ribonuclease Protection Assays (RPA)

RPAs were performed to examine relative levels of transcript for VEGF. All samples were evaluated in a single assay. The cDNA probes for ovine VEGF were graciously provided by Dr. Dale Redmer (North Dakota State University, Fargo, ND) and have been detailed previously [25]. The cDNA probe for the housekeeping gene β-actin, pActn9, was constructed in our laboratory using the following primers: ACC GTG AAA AGA TGA CCC AG, and GCT GTA CCA CAT CTG CTG GA by reverse transcription PCR. The pActn9 sequence was validated by sequence analysis to ensure alignment with the ovine β-actin sequence (accession# AF035422).

The RPAs were conducted as per manufacturer's directions using 20 μg of total mRNA with β-actin used as the housekeeping gene. Radio-labeled [a-^32^P] antisense probes were synthesized by *in vitro *transcription using either T3 or T7 polymerases (Ambion, Austin, TX) in the presence of unlabeled nucleoside triphosphates and gel purified. Following RNase digestion, the hybridization products were ethanol precipitated and separated by electrophoresis. The gel was vacuum dried on to filter paper and exposed to X-ray film. Band sizes were determined with radio-labeled size markers generated with an *in vitro *transcription kit (Ambion, Austin, TX). Bands were visualized, cut from the gel and quantified as counts per minute over a five minute period with a liquid scintillation counter (Beckman Coulter Inc., Fullerton, CA).

### Reverse Transcription Polymerase Chain Reaction Analysis (RT-PCR)

Non-quantitative RT-PCR was performed to determine the splice variant forms of the VEGF transcript present in the fetal LV tissue. The ovine VEGF primers have previously been described [26]. Briefly, 5 μg of total RNA was amplified using the Platinum^® ^SuperScript II Single Step RT-PCR kit (Invitrogen, Carlsbad, CA). PCR amplicons were separated using a 0.7% agarose TBE gel and visualized using an ultra violet light source.

### Real Time RT-PCR

Real time RT-PCR was performed in the manner described previously to assess relative differences of AT1 or AT2 transcript between C and NR fetal LV [24]. In brief, real time RNA samples were amplified using either SYBR^® ^Green technology (AT1) or Taq-Man^® ^technology (AT2) and analyzed using the ΔCT method (User Bulletin #2, Applied Biosystems, Dec 11, 1997) and GAPDH transcript as a normalizer. RT-PCR primers were developed for ovine AT1 (accession# AF069750), ovine AT2 (accession# S81979) and GAPDH (accession# AF35421) sequences, and Taq-Man^® ^probes for Ovine AT2 and GAPDH using Primer Express v2.0 (Applied Biosystems, Foster City, CA) software (Table 1). Primers and probes were synthesized by Applied Biosystems (Foster City, CA). The RT-PCR reactions were prepared using the Platinum^® ^SYBR^® ^Green Quantitative PCR SuperMix-UDG kit (Invitrogen) for AT1 or Platinum^® ^Quantitative PCR SuperMix-UDG kit (Invitrogen) for AT2 as per the manufacturer's directions. Amplification was carried out using a Perkin-Elmer 9600 RT-PCR Machine (Perkin-Elmer) and a 2-step cycling program (95°C for 2 min, and 45 cycles of (95°C for 15 sec, 60°C for 30 sec). RT-PCR was carried out only after the primers had been validated across four different concentrations to demonstrate approximately equal amplification efficiencies.

**Table 1:** PCR primer and Taq-Man^® ^detection probes sequences for interrogation of AT1, AT2, GAPDH by qRT-PCR.

Primer/Probe	Sequence
GAPDH-1	GCA AGT TCC ACG GCA CAG T
GAPDH-2	GGT GAT GGC CTT TCC ATT GA
GAPDH-det	(6FAM)AAG GTC AGA AAA CGG GAA GCT CGC TC(MGBNFQ)
AT2R-1	CAT GGA AGG GAA GCC AAC AA
AT2R-2	ACT CGT GAC CAA GTT CTG AAG ATG
AT2R-det	(VIC)TGA TGA ACG CCA GAA CAA CAG CAG C(MGBNFQ)
AT1U604	CCC TTC GGC AAT TAC CTA
AT1L682	GCG GTC AAT GCT TAG ACA

### Western Blot Analysis

Immunoblots for detection of AT1 (306) and AT2 (H-143) protein was performed as described in detail previously using 50 μg of total LV protein separated using SDS-PAGE [10]. VEGF was detected in the same manner using commercially available polyclonal antibodies VEGF (A-20) obtained from Santa Cruz Biotechnology (Santa Cruz, CA). All samples for each protein of interest were run in a single assay. Immunodetection of proteins was done in the following manner: Membranes were blocked for 1 hour in protein blocking solution (Blotto in TBS, Pierce; Rockford, IL), then incubated in the primary antibody for 1 hour (AT1, AT2, VEGF, all at 1:500) in Blotto TBS blocking buffer (Pierce; Rockford, IL). All dilutions were determined empirically to achieve minimal background. Following the primary incubation, the membrane was washed 4 × 5 minutes each using Pierce western wash (Pierce, Rockford, IL). The membranes were then incubated in the appropriate secondary antibody; goat anti-rabbit IgG conjugated horseradish peroxidase (SantaCruz Biotechnology, Santa Cruz, CA) diluted to 1:1000. Following incubation in the secondary antibody, membranes were washed 4 × 5 min in Pierce western wash (Pierce, Rockford, IL). Blots were detected using a chemiluminescent detection system (Supersignal^® ^West Pico substrate, Pierce; Rockford, IL). Membranes were incubated in chemiluminescent substrate for 5 minutes, and exposed to film (Kodak) from 0.5–20 min as necessary to detect signal. Images were optically scanned (Hewlitt-Packard 5200C with HP PrecisionScan software v2.02) and digitized, and antigen signal was quantified by pixel density (Un Scan It gel v5.1; Silk Scientific, Orem, UT). Quantification was performed only after linearity was established between amount of protein and film exposure time. The membrane was stripped using a commercially available buffer (Restore, Pierce) and re-probed for detection of β-actin at a 1:20,000 dilution (Abcam 276100, Cambridge, UK) for use as a loading control. β-actin was detected as described above with goat anti-mouse IgG HRP diluted to 1:40,000 (Calbiochem 401215; San Diego, CA).

### Data Analysis

All data are presented as mean ± SEM. Differences in the means between the C and NR fetuses were assessed using independent *t*-tests with a Welch correction for unequal variances if required (Prism; GraphPad Software; San Diego, CA). Significance was accepted when P < 0.05.

## Results

### Animals and LV Weights

Consistent with previous reports from fetuses at 78 dGA [6,7], no differences were observed between twin and singleton fetuses in either treatment group, or between male and female fetuses in the control group. Sex difference could not be assessed in the NR group. Data were therefore pooled to comprise the C and NR cohorts. NR fetuses were 30% lighter than respective controls, while LV : fetal weight ratio was increased in the NR fetuses when compared to the C fetuses (Table 2, P < 0.05).

**Table 2:** Weights and left ventricle weights of the cohort of control (C) and nutrient restricted (NR) fetuses evaluated in the present study.

	Fetal heart weight (g)			Organ wt/foetus wt (%)		
	Control (6)	NR (6)	*P	Control (6)	NR (6)	*P

Fetus	335.1 ± 32.4	220.2 ± 10.0	0.004	--	--	--
LV	1.02 ± 0.09	0.91 ± 0.11	NS	0.31 ± 0.03	0.41 ± 0.04	0.03

*P-value control *versus *restricted.

### AT1, AT2, and VEGF mRNA levels

**Figure 1:**
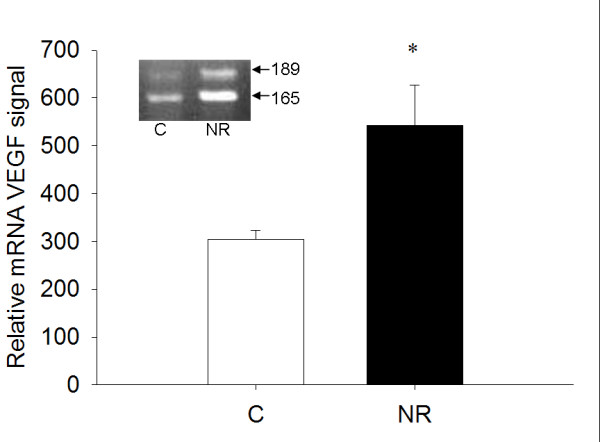
Amount of VEGF mRNA relative to β-actin mRNA in the LV of 78 dGA fetuses as determined by ribonuclease protection assay. Inset picture depicts representative results of RT-PCR reactions for VEGF_165 _and VEGF_189 _run on a 1% agarose gel. NR (■, n = 6) fetuses exhibited increased VEGF transcript in the LV compared to C (□, n = 6) fetuses. *P < 0.05.

AT1 and AT2 mRNA levels were not different between the C and NR LV as assessed by real time RT-PCR (1.099 ± 0.237 vs. 0.827 ± 0.082 and 1.075 ± 0.228 vs. 1.210 ± 0.208). LV tissue from fetuses of NR ewes had greater levels of mRNA for VEGF (Figure 1) than respective C LV when measured by RPA. The RT-PCR of total RNA from fetal LV for VEGF splice variants indicated that VEGF_165 _and VEGF_189 _splice forms are the primary transcripts in the LV tissue at mid-gestation (Figure 1).

### AT1, AT2, and VEGF immunoreactivity

**Figure 2:**
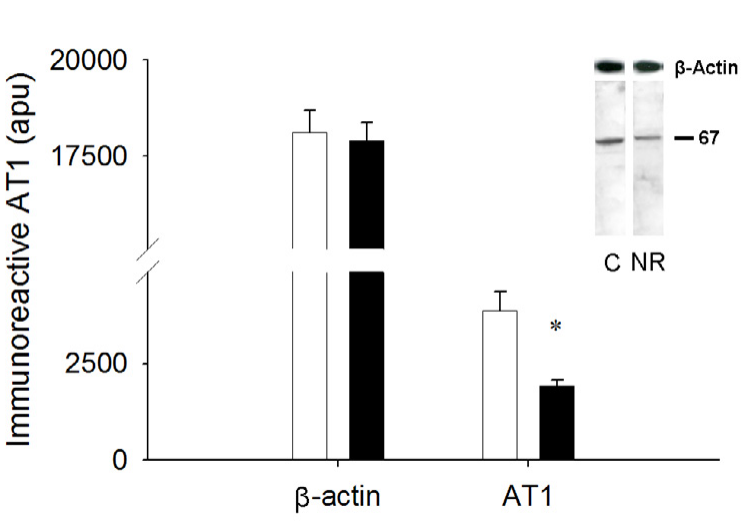
Fetuses from NR (■, n = 6) ewes demonstrated less AT1 expression than C (□, n = 6) LV. Inset depicts representative immunoblot for AT1 and β-actin. Equal protein loading between lanes was demonstrated by re-probing membranes for β-actin. *P < 0.05.

**Figure 3:**
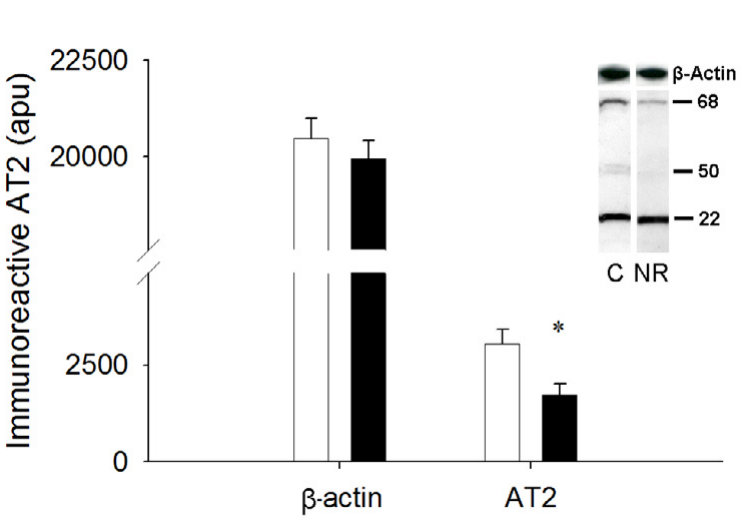
Fetuses derived from NR (■, n = 6) ewes exhibited decreased AT2 expression in the LV compared to fetuses from the C (□, n = 6) ewes. Inset depicts representative immunoblot for AT2 and β-actin. Equal protein loading between lanes was demonstrated by re-probing membranes for β-actin. *P < 0.05.

**Figure 4:**
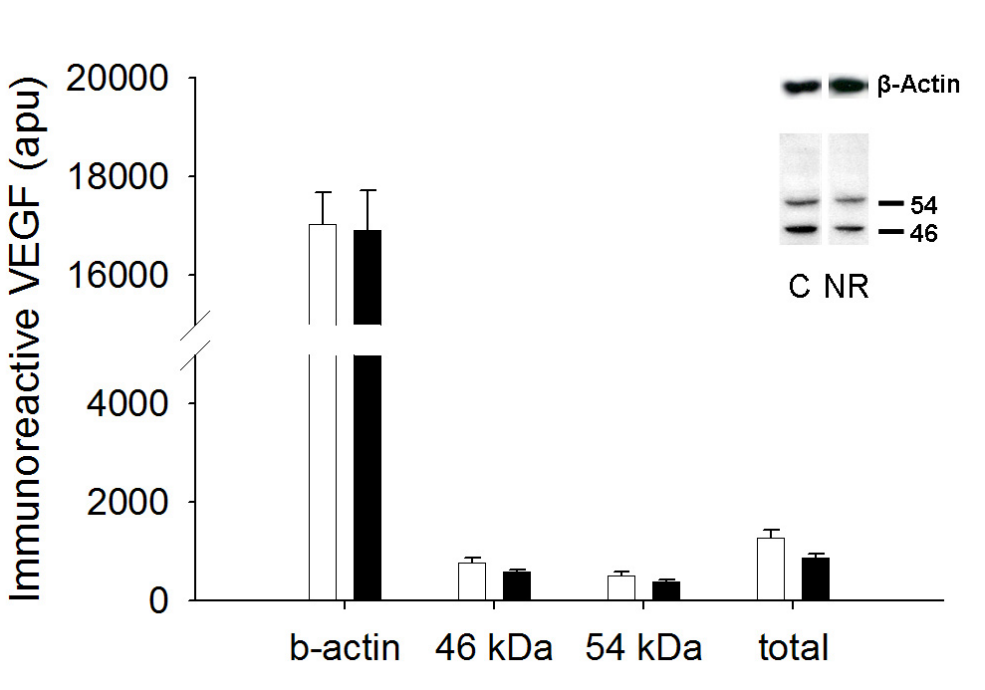
Fetuses derived from NR (■, n = 6) ewes exhibited no difference in total immunoreactive VEGF, or immunoreactive VEGF_165 _(46 kDa) or VEGF_189 _(54 kDa) isoforms in the LV compared to fetuses from the C (□, n = 6) ewes. Inset depicts representative immunoblot for VEGF and β-actin. Equal protein loading between lanes was demonstrated by re-probing membranes for β-actin.

NR LV tissue had less (1918.4 ± 154.2 vs. 3881.2 ± 494.9 apu; *P *< 0.05) immunoreactive AT1 at 67 kDa, previously shown to be the primary immunoreactive band in the ovine fetal heart [18], than respective control fetal LV tissue (Figure 2). The 68 kDa form of AT2, previously shown to be the primary immunoreactive band in ovine coronary and mesenteric arteries [27] was reduced in the NR (1729.9 ± 293.6 vs. 3043.3 ± 373.2 apu; *P *< 0.05) compared to the C fetuses (Figure 3). Smaller immunoreactive bands, the exact identity of which remains unclear, were observed for both AT1 and AT2. No differences were observed between the C and NR groups in these smaller immunoreactive bands (data not shown). These smaller bands could be either the non-glycosylated form that has been reported previously or a degradation product [28]. VEGF was visualized as two immunoreactive bands (46 and 54 kDa) when evaluated under reducing conditions (Figure 4). Total VEGF protein (sum of both bands) was not different between the C and NR LV (1278 ± 164 vs. 860 ± 89 apu; *P *= 0.11). The same result was revealed by further analysis of each band separately (46 kDa, 767 ± 99 *vs. *576 ± 60, *P *= 0.14; 54 kDa, 511 ± 73 *vs. *384 ± 40 apu, *P *= 0.15; Figure 4)

## Discussion

In the present study we have examined cardiac LV tissue obtained from ovine fetuses at 78 dGA generated using an established model of maternal NR-induced growth restriction in which heart weight is protected [6] We have tested the hypothesis that the increase LV mass relative to fetal body weight following global maternal NR during early to mid-gestation is associated with increases in the expression of VEGF and the intra-cardiac renin-angiotensin system receptors. In addition to confirming both the IUGR and preservation of heart mass previously reported, the principle findings of this study are that the LV of NR fetuses demonstrates: 1) decreased AT1 and AT2 protein without a decrease in AT1 and AT2 mRNA; and 2) increased VEGF mRNA without an increase in total VEGF protein; leading to the conclusion that VEGF and the intra-cardiac renin-angiotensin system receptors are not directly involved in the protection of left ventricular mass observed in this model.

The primary objectives in the present work centered on characterizing alterations in expression of specific genes in the fetal LV that are induced by global maternal NR during early to mid-gestation in the fetal sheep. Consequently tissues were captured specifically for this purpose. Recent work has shown nearly all cardiomyocytes are mono-nucleated at mid-gestation, and that conversion to the mature, bi-nucleate state do not begin in earnest until approximately 120 dGA in sheep [29]. A change in cardiomyocyte size appears to be strongly related to conversion to bi-nucleated status and is therefore also a phenomenon of late gestation [29]. Since we were interested in evaluating mid-gestation alterations in the fetal LV that might contribute to persistent changes in heart development and function, we chose to evaluate VEGF and the AngII receptors rather than to perform detailed morphometric analysis at this early stage in myocardial development. Histological evaluation of both the mono-nucleate : bi-nucleate ratio and cardiomyocyte size and volume nevertheless remain an important future objective.

### Angiotensin II Signalling

AngII signaling is involved in cardiac growth, largely through hyperplastic mechanisms in both myocytes and fibroblasts [16,18,19]. Previous work has shown distinct patterns of AT1 and AT2 mRNA and protein expression during fetal heart development in the sheep [14,29]. At the sub-cellular level, AT1 and AT2 mediate effects through disparate means. Whereas AT1 activates multiple signal pathways including calcium, phospholipids, kinases and reactive oxygen species, the AT2 receptor is typically linked to pathways such as phosphatases and protein dephosphorylation, and nitric oxide [30]. That AT1 activates kinases while AT2 activates phosphatases provides a general indication of the present notion that the two receptors are often antagonistic [31].

We show no change in mRNA levels of the AT1 and AT2 receptors. These observations are consistent with the previous report in the same cohort of animals [7] and those of others studying sheep [32]. In contrast, Kijima *et al*. have reported increased expression of AT1 and AT2 mRNA and protein in neonatal rat cardiomyocytes stretched *in vitro *[33]. The present data, viewed in concert with the above studies and the previous work by Edwards showing increased fetal blood pressure in NR fetuses [13], suggests that the nutrient restricted ovine fetal heart may be subject to increased afterload rather than volume overload.

Interestingly, we report that AT1 and AT2 are decreased at the protein level with no change in mRNA expression for these proteins. Previous work has shown AT1 and AT2 mRNA and protein demonstrate negative regulation following AngII exposure *in vitro *at several different levels, including transcriptional and post-translational [34,35]. While little work has been done to evaluate protein expression of AT1 in the fetus, contemporary literature acknowledges the existence of regulatory points for translational and post-translational control of AT1 expression that support a discordance between protein and mRNA for AT1 [36,37]. Neither circulating nor tissue AngII concentrations were measured in the present study, however circulating AngII does not figure prominently in the regulation of the cardiac AngII receptors since cardiac interstitial AngII is typically over 100 fold higher in concentration than plasma AngII [38]. Consequently, the present observations may indicate that chronically augmented intra-cardiac AngII production exists in the LV of the NR fetus since receptor protein expression was inhibited in this group compared to the C group.

The decrease in immunoreactive AT1 suggests that regardless of the type of growth occurring (i.e. hyperplasia, hypertrophy or extracellular matrix proliferation), alternative mechanisms to AT1 signaled growth must be operative in the NR fetus. Previous work has shown that, when AT1 receptors are absent, hypertrophic stimuli act via induction of tyrosine kinase pathways [39]. This observation may explain why *in vivo *blockade of AT1 receptors during pressure overload does not completely attenuate ventricular hypertrophy in the late gestation fetal sheep [18]. Similarly, the present findings are consistent with previous work showing that hypertrophic responses in neonatal cardiomyocytes induced by mechanical stretch are not ablated by AT1 inhibition [40]. Alternatively, in light of the recent work by Sundgren *et al*. [16], the observed decrease of AT1 in the NR LV may result in reduced proliferation of cardiomyocytes and consequently an impaired myocyte endowment in the NR animal, thereby contributing to reduce cardiovascular function and reserve in later life [41,42].

Although the actions of AT2 in the fetal heart remain nebulous, present evidence indicates AT2 regulates cell growth in cultured fetal cardiomyocytes [43]. AT2 is also recognized as a promoter of vasodilation [44]. The observed reduction of AT2 in the NR heart may therefore represent a development deficit in vasodilatory capacity and/or accelerated maturation of the coronary circulation. This is in part consistent with observations by Nishina *et al*. who reported impaired vasodilatory responses in femoral resistance arteries from undernourished sheep fetuses at mid-gestation [45]. Unfortunately, the limited tissue availability from the present study did not allow for receptor localization via immunohistochemistry. As with the AT1 receptor, we report a discord between gene transcription and immunoreactive AT2 in the NR fetal LV. Recent work examining the kidneys of offspring from protein restricted rat dams shows AT2 mRNA increases while AT2 protein expression is decreased; however no mechanisms have been described or proposed for this observation [46]. Clearly, further work is needed to thoroughly investigate these observations.

### Vascular Endothelial Growth Factor

Because of the observed preservation of LV mass in the face of IUGR, one might suspect an increase in VEGF expression to provide adequate vascularity to the augmented cardiac tissue mass. VEGF is thought to be involved in compensatory coronary vascular growth since its expression is increased in the hypertrophied hearts of spontaneously hypertensive rats and in experimental models of pressure overload [47,48]. The existence of heparin binding domains alters VEGF solubility and has profound impacts upon its activity/function. VEGF_165 _contains 1 heparin binding region and therefore has limited solubility, while VEGF_189 _is not freely diffusible and binds tightly to the extra cellular matrix [49]. We have shown that both VEGF_165 _and VEGF_189 _protein were confirmed present using isoform specific RT-PCR. Since VEGF_165 _has limited solubility and VEGF_189 _is not soluble, it is not likely that the observed discordance between mRNA and immunoreactive protein is a consequence of translated VEGF being secreted and transported away from the heart in the circulation. Moreover, we have previously reported in sheep that fetal plasma VEGF at mid-gestation is not altered by maternal NR [6]. Finally, the discordance between VEGF transcription and translation reported in this study agrees with previous data by others in adult rat cardiac tissue [50]. Furthermore, acute increases in afterload in the fetal sheep by means of a 7 day infusion of Ang II results in ventricle hypertrophy without increases in VEGF mRNA expression [19]. In fact, these authors reported that phenylephrine infusion decreased VEGF mRNA in the fetal LV and RV while ventricular mass increased. Unfortunately protein expression was not investigated. Nonetheless, these data collectively suggest that VEGF is not directly involved in preserving LV mass relative to fetal body weight at mid-gestation following maternal NR.

We have previously identified an increase in gene transcription for a recently described neuropilin-1 like protein [51], endothelium derived smooth muscle neuropilin (ESDN), in the NR fetal LV [7]. Neuropilin-1 has been found to facilitate VEGF_165 _signaling. Since the activities of ESDN have not been fully characterized, at least to our knowledge, we cannot rule out the possibility that ESDN may possess neuropilin-1 like properties. If ESDN protein is increased along with gene transcription, VEGF_165 _activity may be increased without a concomitant increase in protein. Without further insight into the function of ESDN as it relates to VEGF, we interpret the present findings to mean that VEGF protein expression is not intimately involved in the observed preservation of ventricular weight. We further suggest that this observation may allow for the development of a vascular deficit in the fetal myocardium following NR.

### VEGF and the Intracardiac Renin-Angiotensin System

Previous investigators have described an interaction between the renin-angiotensin system and VEGF systems in several adult animal species. Recent work has demonstrated that Ang II exerts pro-angiogenic effects via the AT1 receptor and VEGF in mice [52], while AT1 inhibition is shown to impair VEGF mediated coronary angiogenesis in hamsters [23]. Similarly, Sarlos *et al*. have shown that AT2 interacts with VEGF in the promotion of angiogenesis in the fetal rat retina [53]. The lack of change in VEGF protein in the present study may therefore be a result of the reduced expression of AT1 and/or AT2 in the fetal LV. Since evaluation of the systems that lie downstream of AT1 and AT2 is beyond the means of the present work, further studies are necessary to determine if this is indeed the case in the present model.

## Limitations

The present study has several limitations. The origin of the protection of cardiac LV mass (i.e. hypertrophy, hyperplasia or extracellular matrix) remains unclear and further experiments should be undertaken to evaluate this properly. With respect to the observed discordance between gene transcription and protein expression, it should be noted that one cannot discount the possibility that novel regulatory mechanisms may be at work in the fetus when compared to the adult. Interrogation of the discord is unfortunately beyond the scope of the present work and tissue availability at this time. Finally, it remains unclear whether differences between male and female fetuses exist during normal fetal cardiac development, and whether sex differences may contribute to the present differences in the gene transcription and protein expression in response to maternal NR in this study.

## Conclusion

We have demonstrated decreased immunoreactive AT1 and AT2 receptor protein without a decrease in mRNA, and increased VEGF mRNA with no change in VEGF protein, in the fetal LV at mid-gestation. As a result of these findings, we reject the hypothesis that VEGF and the renin-angiotensin system [via AT1 and AT2] are primary mediators of the observed protection of LV mass in the NR fetuses. Furthermore, these data show no evidence of a compensatory increase in VEGF protein expression that would be expected concomitant with the augmented LV : fetal weight ratio. We postulate that the mechanisms underlying the discord between gene transcription and protein expression for both VEGF and AT1/AT2 may involve decreased translational efficiency or increased protein turnover. The present findings may contribute an important first step towards elucidating mechanisms underlying epidemiological studies that indicate fetuses with low birth weight carry an increased risk of mortality from cardiovascular disease, particularly if these individuals have reduced cardiovascular reserve due to an epigenetic decrease in vascularization. While these results are intriguing, the scope of these findings is narrow and further experiments are needed to place these data into perspective.

## Authors' contributions

JSG drafted the manuscript, performed statistical analyses, participated in the tissue collection and contributed to the molecular biology analyses. ALL designed and validated RT-PCR primers, and contributed to the molecular biology analyses and critically reviewed the manuscript. MJN designed the study and participated in the tissue collection, data interpretation and draft of the manuscript.

## Abbreviations

AngII = angiotensin II; AT1 = AngII receptor type 1; AT2 = AngII receptor type 2; C = control; dGA = days of gestation; LV = cardiac left ventricle; NR = nutrient restriction; RNA = ribonucleic acid; RPA = RNA protection assay; RT-PCR = reverse transcription polymerase chain reaction; VEGF = vascular endothelial growth factor; IUGR = intrauterine growth restriction; apu = arbitrary pixel units
